# With whom do researchers collaborate and why? 

**DOI:** 10.1007/s11192-017-2386-y

**Published:** 2017-04-13

**Authors:** Hajdeja Iglič, Patrick Doreian, Luka Kronegger, Anuška Ferligoj

**Affiliations:** 1 University of Ljubljana, Ljubljana, Slovenia; 2 University of Pittsburgh, Pittsburgh, USA

**Keywords:** Scientific collaboration, Collaboration levels, International collaboration, Collaboration with industry, Biotechnology, Mathematics, Physics, Sociology

## Abstract

Although research collaboration has been studied extensively, we still lack understanding regarding the factors stimulating researchers to collaborate with different kinds of research partners including members of the same research center or group, researchers from the same organization, researchers from other academic and non-academic organizations as well as international partners. Here, we provide an explanation of the emergence of diverse collaborative ties. The theoretical framework used for understanding research collaboration couples *scientific and technical human capital* embodied in the individual with the *social organization* and *cognitive characteristics* of the research field. We analyze survey data collected from Slovenian scientists in four scientific disciplines: mathematics; physics; biotechnology; and sociology. The results show that while individual characteristics and resources are among the strongest predictors of collaboration, very different mechanisms underlie collaboration with different kinds of partners. International collaboration is particularly important for the researchers in small national science systems. Collaboration with colleagues from various domestic organizations presents a vehicle for resource mobilization. Within organizations collaboration reflects the elaborated division of labor in the laboratories and high level of competition between different research groups. These results hold practical implications for policymakers interested in promoting quality research.

## Introduction

Knowledge production in the twentieth century was characterized by a steady rise in the scale and importance of scientific collaboration. Although science has always been a social rather than a solitary enterprise due to the need to share ideas and validate scientific findings with colleagues (Finholt and Olson 1997), various social, economic, technological, and cognitive changes created an unprecedented level of research cooperation (Bozeman and Boardman 2014).

Recent explanations of the ever-increasing research collaboration suggest it is driven by the growing number of scientists applying for research funds (O’Brien 2012). This contributed to greater competitiveness and specialization at the individual level (Wenger 1998; Blau 1994). In such highly competitive research environments, increased specialization puts pressure on scientists to cooperate with colleagues possessing complementary skills and knowledge. When looking for partners, they often consider those having high prominence and greater scientific productivity (Crane 1972; Beaver and Rosen 1978, 1979a, b) who can help them gain access to scarce resources. This era has been characterized by Big Science in which the scale and comprehensiveness of research projects have increased (Price 1963; Galison and Hevly 1992) increasing the resource dependencies between scientists. Equally important, science made the transition from the Mode l to the Mode 2 type of knowledge production where multi-, inter- and trans-disciplinary teams were formed to work on very applied or real-life problems (Gibbons et al. 1994). The recent shift from an Industrial to a Knowledge Society implies also increased triadic cooperative relationships involving academia, industry, and government, known as the Triple Helix thesis (Etzkowitz and Leydesdorff 1995, 2000).

Further, advances in information and communication technologies enabled collaboration between geographically dispersed research units increasing the incidence of more successful research (Kouzes et al. 1996; Finholt 2002; Atkins et al. 2003; Hara et al. 2003; Nentwich 2003). Policy leaders and academic managers also encourage research collaboration crossing disciplinary, organizational, sectorial, and national boundaries by employing the co-opetition strategy (Brandenburger and Nalebuff 2011), understood as “cooperation between competitors” to improve the competitive advantage of research units and stimulate economic growth (Sonnenwald 2007). The best results for institutions and individuals are thought to be achieved not solely through competition but also through careful collaboration stimulated in multiple ways: financing and evaluating research work with a focus on research groups and institutions rather than on individuals; having funding agencies impose conditions for acquiring research funds including imposing minimum sizes for research groups; by requiring research groups to work with teams from different institutions, sectors, disciplines, and countries; emphasizing large-scale funding rather that smaller grants; and supporting applied research topics rather than theoretically-oriented research. Together with stimulating collaboration, policy leaders and academic managers intensify their role in setting the standards of quality through the elaborated evaluation processes and reward structures.

Patterns of individual-level collaboration express researchers’ choices for working together in the context of contemporary science processes characterizing the last decades of the twentieth century and continuing into the twenty-first Century. Our model of collaboration includes the scientific and technical human capital theory (Bozeman et al. 2001) and a resource-based view on collaboration (Van Rijnsoever et al. 2008). Both stress the role of resources embodied in individuals and assume that individuals undertake collaboration to enhance their human capital (Bozeman and Boardman 2014). We extend this model to capture also the features of the changing social organization of the research fields and their cognitive (intellectual) characteristics (Birnholtz 2007), and argue that individual collaborative strategies are promoted or hindered by these contextual factors.

Van Rijnsoever et al. (2008) suggested factors often associated with collaboration levels have different impacts in distinct types of collaborative networks. It is important to examine the factors promoting collaboration with different types of partners. Since our study is of a small national science system, we anticipate factors explaining international collaboration differ considerably from those accounting for domestic collaboration. In small science systems, international collaboration allows researchers to specialize and connect with partners holding complementary knowledge unavailable inside the country. It also allows scientists from smaller and less central science systems to connect with global knowledge production centers and with more prominent researchers. In the domestic arena, the primary distinction is between intra- and inter-organizational collaboration. We anticipate inter-organizational collaboration being a vehicle for resource mobilization. Researchers turn to colleagues in other organizations to gain access to additional resources and to apply jointly for research funds from national research agencies. In contrast, strong intra-organizational collaboration is expected to relate to the elaborated division of labor, especially in laboratories, and highly competitive research environments.

In summary, the major aim of this study is to understand scientific collaboration between *different types of research partners* by using three groups of factors: *human capital* of individual researcher, *cognitive aspects* of knowledge production and the *social organization* of science. The results are informative regarding which kinds of collaboration are stimulated by the major changes in contemporary knowledge production including the increased competition for resources and prestige, specialization of scientific knowledge, intensification of resource dependence, greater emphasis on interdisciplinary, innovative and applicable knowledge, and standardization of the criteria regarding quality research.

While this study is limited to the Slovenian science system, we assume researchers there—as elsewhere—work in very different cognitive and social contexts given their specific fields of research.

## The empirical context: Slovenian science

The Slovenian scientific research system is small. About 15,000 persons are involved, among them 8500 researchers (measured in Full Time Equivalents, FTEs) who are employed in 42 higher education institutions, 47 research institutes and 777 business units registered for conducting research and development (Udovič et al. 2016). Trends in the number of researchers, gross domestic expenditure on R&D and bibliometric data reveal the high level of dynamism characterizing Slovenian science after the political transition in the early 1990s. The number of researchers increased by about 50% in the last 10 years. Gross domestic expenditures on R&D, as a percentage of GDP, increased between 1996 and 2015 from 1.33 to 2.39%, with business R&D expenditure accounting for about three quarters of the total R&D (Udovič et al. 2016). Both the number of researchers per million inhabitants and the overall R&D intensity are comparatively high, especially compared to other Central and Eastern European countries (OECD 2012).

The public financing of research programs and projects is highly centralized and competitive. Slovenian research policies placed great emphasis on increasing publications and their impact over the last decades. Currently, Slovenia ranks high among the EU members for the rate of growth of the number of publications and the growth of received citations. Among the four research fields included in our study, physics and mathematics achieved above-average impact factors by 2012. Biotechnology changed from being primarily domestic to involving international publications. The number of its received citations tripled with its impact factor doubling. The number of international publications and citations also increased in sociology. However, domestic publications still outstrip international ones in this field (Sorčan et al. 2008). Despite these improved performances, the share of Slovenian scientific publications among top 10% most cited remains below the EU average (Hollanders et al. 2016).

The rise in the number of domestic and international publications, along with increased citations, was accompanied by increasing scientific collaboration. According to the European Innovation Scoreboard (Hollanders et al. 2016), Slovenia’s performance is especially strong regarding collaborations involving international scientific co-publications, public–private scientific co-publications and innovative SME’s collaborating with others. Various Slovene research policies aimed at strengthening the international and domestic collaborative potential of Slovenian science, varied from supporting the involvement of researchers into the EU research projects to financing bilateral collaboration, establishing centers of excellence and centers of competence (Udovič et al. 2016). The latter are specifically targeted at strengthening long-term cooperation between the public research and business spheres, and of interdisciplinary research (Stare et al. 2014). When applying for public funds to support basic research, researchers must collaborate with colleagues from at least one other organization. When they apply for grants supporting applied research they should collaborate with non-academic partners contributing their share of funds.

An important part of the steep increase in research collaboration by Slovenian researchers involves collaboration with researchers outside Slovenia. A recent review in Knowledge, Networks and Nations (Wilsdon 2011) indicates that for 1996–2000, Slovenian researchers collaborated most intensely with colleagues from Germany, while in the period 2004–2008 they expanded collaboration to include also Italy and France. This is consistent with findings showing the collaboration patterns of smaller countries when their researchers tend to cooperate with colleagues from selected larger countries rather than across all of Europe (Frenken 2002; Frenken and Leydesdorff 2004; Ukrainski et al. 2014).

Bibliometric data show the number of co-authored publications grew significantly faster than the number of solo publications in all four scientific disciplines included in this study. In the period from 1986 to 2005: the incidence of solo publications in sociology dropped from 70 to 30%; in mathematics from 65 to 30%; in physics from 16 to 11%; and in biotechnology from 17 to 4% (Kronegger 2011). Together, the increased number of researchers, strong competition for funds, especially among researchers depending on public R&D funds, strong emphases on academic excellence and international prominence, and research policies favoring collaboration across disciplines and sectors, contributed to the trend towards increased number of co-authored publications. Clearly, Slovenian science is representative of modern day science.

## Hypotheses

The Scientific and Technical Human Capital theory (STHC), formulated by Bozeman et al. (2001), states that individuals bring unique sets of resources to their work and collaborative efforts such as formal education and training, research experience, knowledge, skills and reputation, as well as social ties with a variety of actors. The STHC theory assumes researchers engage in collaboration to enhance their human capital. Empirical studies of motives for collaboration confirmed researchers as viewing collaboration strategically to create new synergies in knowledge and skills, increase the number and visibility of their publications, and improve the professional prestige (Melin 2000; see Bozeman and Corley 2004 for a review). In the resource-based perspective on collaboration (Van Rijnsoever et al. 2008) it is further assumed that collaborating with colleagues in different types of organizations (e.g. university research centers, private companies, international partners) enhances different aspects of human capital.

While building on STHC theory and resource-based perspective, we examine also the contextual features of science including both cognitive and social dimensions of research fields (Birnholtz 2007) facilitating or hindering researchers' efforts to improve their STHC through collaboration. Although the literature provides a long list of social and cognitive aspects of the research contexts that are relevant for collaboration, we focus only on those reflecting the changing nature of contemporary knowledge production: competition-related secrecy and *distrust* between the scientists; the *need for professional help* from colleagues due to greater specialization of knowledge; *resource dependence* resulting from skyrocketing costs of research; *interdisciplinary composition* of research teams as answers to demands for innovative knowledge; the promotion of *standardized criteria of quality* as enforced by rewards structures and evaluation processes.

To examine the extent of research collaboration involving different types of research partners, we organize predictor variables into three sets: (1) individuals’ human capital; (2) cognitive aspects of research fields; and (3) social aspects of these fields. Our hypotheses deal with the relationships between the three sets of predictor variables and research collaboration. As the hypotheses for the same outcome can be viewed as either complementary or rival hypotheses, they were all tested while controlling for other predictor variables.

### Individual human capital

This set of predictor variables includes *research experience* and *career advancement*.

Research experience is individual endowments of human and social capital separate from, while influenced by, their research environments. The longer researchers engage in research, the more knowledge and skills they accumulate. Furthermore, the larger the number of potential collaborators, since engaging in past collaborations, the greater the access to social capital useful for engaging in future research projects. Yet the relationship between research experience and collaboration is nonlinear. Van Rijnsoever et al. (2008) found that after approximately 20 years of an active research career, collaborative activity starts decreasing. The resulting inverted U-shaped relationship between research experience and collaborations must be included in any analysis of collaboration. One argument for this downward turn is that experienced researchers have accumulated enough human capital—knowledge and skills—permitting them greater freedom for solo research. Another claim is that older researchers are more likely to occupy administrative and other engagements, leaving them with less time for extensive collaborations (Van Rijnsoever et al. 2008). It has also been shown that younger and mid-career scientists have greater productivity pay off from collaboration than older researchers (Lee and Bozeman 2005). This leads to:

#### **Hypothesis 1**

The relationship between research experience and level of collaboration takes an inverted U-shape.

Administrators and managers set requirements for professional and academic promotion. These requirements demand the production of greater quantities of work having high visibility to advance a researcher’s academic career (Ponomariov and Boardman 2010). Collaborating with others leads to higher quality academic work which opens the doors for publishing in international journals and enhances citation-related productivity (Katz and Hicks 1997; Lee and Bozeman 2005; Persson 2010). Also in university–industry collaboration there is a strong tendency toward publications-focused outputs (Ambos et al. 2008; Levy et al. 2009). Yet, recent studies have found the positive impact of collaboration on academic careers to be limited to academic collaboration. Collaboration with colleagues from industry and interdisciplinary collaboration can have negative impacts on the careers of academic researchers (Van Rijnsoever and Hessels 2011). Academic reward structures appear to be designed to favor monodisciplinary research (De Boer et al. 2006) since monodisciplinary papers tend to receive more citations than interdisciplinary papers (Levitt and Thelwall 2008). For researchers in small science systems, collaboration with international academic partners is especially rewarding when their papers are published in more prestigious journals and receive more citations (Kyvik and Larsen 1997). This suggests:

#### **Hypothesis 2**

The more researchers see collaboration as a means for career advancement the more they collaborate, especially with international partners.

### Cognitive aspects of research fields

Among the predictors capturing cognitive aspects of research fields are *specialization of knowledge*, the prevalence of *common quality standards* between the researchers, and the *composition* of the research teams.

The specialization of knowledge is considered as one of the prime reasons for increased collaboration in contemporary science (Wenger 1998). Scientists encountering research problems unresolvable using their highly-specialized knowledge and skills, need to collaborate with others outside their specialty. Yet there are concerns regarding specialization as fostering deep divisions in science and how this serves to encourage scientists to work with colleagues in their own specialty area. One study of collaboration patterns in sociology identified three collaboration patterns confirming the predominance of within-specialty collaboration over engaging in complementary collaboration across different intellectual terrain styles (Leahey and Reikowsky 2008). Studies comparing large and small national scientific systems have pointed to some disadvantages for small countries. In Slovenia and in other small countries where the domestic scientific community is too small to permit higher levels of internal specialization, researchers turn to international colleagues for help via collaboration (Narin and Whitlow 1990; Luukkonen et al. 1992). This suggests:

#### **Hypothesis 3**

The more often researchers encounter research problems unresolvable using their own knowledge and skills, the more they seek collaboration with other researchers, especially international partners.

Even though researchers could find help from colleagues to be very useful, this does not guarantee collaborations are realized, especially in the presence of serious obstacles to effective communication between potential collaborators. Consistent with Birnholtz (2007), we expect agreement between scientists on the standards for quality scientific work to affect positively collaboration. Agreement on quality work eliminates particularistic judgments, often sources of uncertainty, uneasiness, and interpersonal conflict. This suggests:

#### **Hypothesis 4**

Agreement on the quality of research work creates higher levels of collaboration.

The complex research questions scientists work on today require innovative and comprehensive answers compelling them to collaborate across scientific disciplines. Interdisciplinary research can be understood as “the integration of disciplines within the research environment”? (Qin et al. 1997) which positively contributes to knowledge production and innovativeness (Gibbons et al. 1994; Schmickl and Kieser 2008). To produce innovative knowledge scientists are encouraged by funding agencies and administrators to engage in multi-discipline university research centers, university–industry partnerships and centers of excellence, and industry interdisciplinary research collaboration (Bozeman and Corley 2004; Bozeman and Boardman 2014; Cummings and Kiesler 2005; Corley et al. 2006). Thus, the focus on interdisciplinarity widens collaboration patterns to include colleagues from other domestic organizations, academic and non-academic. This leads to:

#### **Hypothesis 5**

Researchers involved in interdisciplinary research projects are more likely to collaborate across the organizational boundaries.

### Social aspects of research fields

The social organization of research includes the *competition-related distrust*, and the relationship of *resource dependence* between the researchers.

Many studies confirm trust as correlating positively with collaborative behavior and the creation of new collaborative ties (Hara et al. 2003; Sonnenwald 2007; Maglaughlin and Sonnenwald 2005). As in other social contexts, expecting others to misuse collaborative relationships by appropriating ideas and research results creates strong disincentives for engaging in collaborative relationships. The highly competitive nature of science potentially reduced collaboration by lowering incentives for sharing research results with competitors and increasing secrecy (Walsh and Hong 2003). But, as noted by Birnholtz (2007), competition does not necessarily reduce collaboration as much as it constrains the set of possible collaborators to close and known colleagues. This leads to:

#### **Hypothesis 6**

Researchers having less trust in colleagues outside their research unit collaborate less, and when they do collaborate they are more likely to engage with researchers within their research units.

Resource dependence theory, initially developed to explain ties between firms (Pfeffer and Salancik 1978; Hagedoorn 2002) engaging in collaboration to build competitive advantage, reduce uncertainty, lower the costs and realize economies of scale, also has been used to explain collaboration between non-firm research organizations (Barney 1991; Grant 1996; Galison 1997). Researchers collaborate when the costs of doing research related to equipment, materials, infrastructures, field work, etc. are too high to be carried by a single individual or research group. Such mutual dependence is found in research fields where resources are scarce and concentrated (Fuchs 1992; Whitley 1984) and in smaller science systems having limited resources for conducting research. This suggests:

#### **Hypothesis 7**

The more scientists depend on pooling resources to conduct research, the more time they spend collaborating with researchers in other academic and non-academic organizations.

### Control variables

Several other factors shown in other studies to affect collaboration were included as control variables. Among them are *scientific disciplines, gender*, and *prior experience* with collaboration.

Scientific disciplines usually show significant impacts on collaboration but only in bivariate analysis: these effects diminish or vanish when other predictor variables are included. Prior effective collaborative experiences were consistently related positively to interest in future collaborations (Birnholtz 2007; Toral et al. 2011). Regarding the effect of gender on collaboration, studies showed women had smaller or similar sized collaboration networks as men (Cameron 1978; Cole and Zuckerman 1984; Bozeman and Corley 2004; McDowell et al. 2006; Van Rijnsoever et al. 2008). Female researchers are more involved in boundary-spanning collaborations involving industry-based researchers (Bozeman and Gaughan 2011) and engage more with interdisciplinary research collaboration efforts (Rhoten and Pfirman 2007; Van Rijnsoever and Hessels 2011), but they have fewer international collaborators than men (Rosenfeld and Jones 1987).

## An empirical study

This study is part of a larger research project on scientific collaboration of Slovenian researchers combining survey and bibliographical data to examine the role of research networks for understanding research collaboration and scientific production. Some results using bibliographical data appeared in Kronegger et al. (2011, 2012), Ferligoj et al. (2015) and Cugmas et al. (2016). Here, we focus exclusively on the findings of a web survey conducted among researchers from four different disciplines: mathematics, physics, biotechnology, and sociology.

The targeted population included all researchers from these scientific disciplines registered in mid-2008 in the SICRIS (Slovenian Current Research Information System) database. They include researchers from universities, public and private research centers, and business firms. The researchers’ disciplinary boundaries are not unambiguously defined as the information system ascribes to each researcher at most two fields. Here, we used only the first listed identification as the primary one.

The questionnaire was sent out at the end of 2010 to researchers using 662 email addresses. After two reminders, the response rate was 52%. However, the population structure and the realized sample correspond quite closely (Table 1). The four fields differ with physicists forming the largest field. Biotechnologists, working in a new emerging techno-science, were the smallest group. Women comprise one-third of this population. The average research experience of these scientists, defined as the time since they published their first professional article, is 19 years.

**Table 1:** Data regarding the realized sample

	All researchers in the field		Realized sample	
	*N*	%	*N*	%
**Disciplines**				
Mathematics	167	25	84	25
Sociology	120	18	92	27
Physics	260	39	106	32
Biotechnology	115	17	54	16
**Gender**				
Women	225	34	130	38
Men	437	66	213	62
**Research experience**	18 years		19 years	
Total	662	100	343	52

Despite having limited resources, our aim was to include a broad spectrum of scientific disciplines to include natural, formal and social sciences. Also, we wanted to include older well-established disciplines and new scientific fields. Disciplines vary by having different emphases on team-work. We also wanted to include basic and applied sciences. Biotechnology is a new and rapidly developing scientific field characterized by research collaboration among researchers from various academic backgrounds. It features collaboration between universities, public research institutions and industry (Heimeriks 2012). Mathematics and physics are mature and relatively stable scientific disciplines with high levels of functional dependence between scientists who must refer to research results of their colleagues to be recognized as competent. Sciences with high functional dependence usually exhibit a strong sense of community and identity clear boundaries with other fields. Mutual dependence between researchers in physics is further reinforced by strategic dependence making this scientific field more competitive and stratified (Whitley 1984). In contrast, sociology is considered a weakly bounded scientific field, with lower levels of mutual dependence, a weaker sense of identity with less agreement regarding research goals, and a less hierarchical social order than in physics.

Yet there has been some convergence between scientific disciplines due to changes in the knowledge production in recent decades. Becher and Trowler (2001) claim most scientific disciplines have moved “beyond the basic-applied dichotomy” with research fields within scientific disciplines exhibiting various levels of applicability. However, the scientific disciplines included in our study remain different in some important aspects: the need for professional help from colleagues is the highest in mathematics and the lowest in sociology. Agreement regarding the standards of quality research is lowest in sociology and the highest in mathematics. Resource dependence is the highest in both physics and biotechnology, lower in sociology and the lowest in mathematics. Interdisciplinary research environments are most common in biotechnology and least common in mathematics.

## Measurement

Katz and Martin (1997: 7) defined collaboration as “working together of individuals to achieve a common goal”. If so, many different people including technicians, administrative staff, and research assistants collaborate in research projects at different points in time. Yet their collaborative roles are not always publicly recognized in the authors’ list or in the acknowledgements. Sometimes, authors are listed in a publication for non-academic reasons (Hagstrom 1965). These deficiencies led to the recognition that co-authorship, a widely-used measure of collaboration since the pioneering works of Price (1963), despite its many advantages including high reliability and comparability of data, has limitations when studying different scientific disciplines where different practices developed and persisted regarding attributions of authorship. This implies in-depth interviews and surveys have value also. Their key feature is measuring collaboration by obtaining information from researchers who are asked to describe aspects of their collaborations with others (Melin 2000; Hackett 2005; Van Rijnsoever et al. 2008; Van Rijnsoever and Hessels 2011; Lewis et al. 2012). This approach was adopted here.

### Predicted variables

We measured collaboration by asking respondents what percentage of their research work in the last 12 months was done: (a) individually; (b) in collaboration with immediate colleagues from the same research unit; (c) with colleagues from the same research organization in different unit; (d) with researchers from other academic organizations in Slovenia (universities and research institutes); (e) with researchers from industry and public sector organizations; (f) with researchers from abroad; and (g) others. This is a slightly modified version of the survey question used by Lee and Bozeman (2005) who studied the impact of collaboration on scientific productivity. The question was posed this way to not introduce an a priori understanding of collaboration. Researchers could define the boundaries of collaborative relations and decide what counts as collaboration. Also, the overall measure *extent of collaboration* was constructed by summing the percentages of time spent collaborating with others, regardless of where the collaborators were located.

Descriptive analyses for all predicted variables—collaborating in general and with different kinds of partners—are provided in “Appendix 1”. Two predicted variables—collaborating with colleagues from academic and non-academic institutions—required a logarithmic transformation for the regression analyses since their distributions are right-skewed.

### Predictor variables

The predictor variables were operationalized by using the following questions to garner information:

- Needing help of other colleagues as an indicator of knowledge specialization: “Would you say that in your research work you often encounter a problem for which you need the support and advice of your colleagues?”
- Agreement about quality work was measured with two questions: “Would you say that, when you assess the work of your colleagues, you usually agree with the evaluations of others?” and “Would you say that, when other colleagues assess your work, you usually agree with their evaluations?”
- Codification of scientific knowledge: “Would you say that most colleagues use the same approaches and research methods while doing research?”
- Trust in researchers outside one’s own research group: “Would you say that in your field you can freely discuss work with colleagues outside your research group without being concerned they would appropriate the results of your research group?”
- Resource dependence: “Would you say that in your field, collaboration is necessary due to the need to share equipment and resources?”
- Contribution of collaboration to career advancement: “Would you say that collaborating with others (could) benefits your career?”
- Prior experience with collaboration: “What is your experience with different aspects of previous collaboration: (a) division of labor in the group; (b) coordination of research work; (c) composition of the research group; and (d) attribution of authorship?”
- Composition of the project team: “Now please think about the co-authored academic work you have written during the last five years which you are especially proud of. Please tell us what was the composition of the research team. Was it: (a) disciplinary and very homogeneous; (b) disciplinary but heterogeneous involving different sub-disciplines; (c) interdisciplinary with moderate heterogeneity, (d) interdisciplinary with strong heterogeneity?”
- Research experience: the number of years since the publication of the first scientific work.

Some predictor variables were developed specifically for this study, others were borrowed from other authors (Van de Ven et al. 1976; Walsh and Hong 2003; Birnholtz 2007). Most answers were measured on a scale from 1-strongly disagree to 5-strongly agree. Prior experience had a scale ranging from 1-very bad to 5-very good. Composition of the research group was recoded into 1- disciplinary, homogeneous; 2-disciplinary, heterogeneous; 3-interdisciplinary. When more than one question was posed for a specific concept, the average score was used. Correlation matrix for predictor variables is provided in “Appendix 2”.

## Results

Our data show researchers spend about 40% of their research time working alone and 60% in collaboration with others (see Table 2). Differences in collaboration between the four scientific disciplines are small but statistically significant. Mathematics is the lowest and biotechnology the highest.

While the ranking of disciplines is similar when collaboration is studied using bibliometric and survey data, variations between them are much smaller in survey data, suggesting mathematics and sociology are also ’social’ fields despite this not being seen clearly in the multiple co-authorships. Using co-authorships as an indicator of collaboration underestimates the presence of ’less visible and invisible forms’ of collaboration in some disciplinary fields (Cronin et al. 2003, 2004).

**Table 2:** Comparing the extent of collaboration and co-authorship

	All	Mathematics	Sociology	Physics	Biotechnology
**Collaboration measures**					
Extent of collaboration	61.95	55.82	59.53	66.27	66.73
Co-authored publications	74.95	58.23	67.11	89.25	85.10
*N*	318	79	85	102	52

Extent of collaboration measures the percentage of research time spent collaborating with other researchers. Co-authorship of publications measures the percentage of co-authored publications among all publications. The number of respondents in different disciplines (*N*) is smaller than in Table 1 due to having a few item-specific instances of missing data

### Explaining the extent of collaboration

The results of regression analyses regarding the prediction of collaboration are reported in Table 3. The reported coefficients are standardized to compare the relative contributions of the predictors. We include in the regression model both research experience and its squared version to capture the inverted U-shape effect of research experience on collaboration.

Model 1 reports only differences by disciplines in the levels of collaboration. Mathematics was used as the comparison base to avoid exact collinearity. Researchers working in physics and biotechnology have significantly higher levels of collaboration than sociologists and mathematicians. However, these differences vanish with the inclusion of other predictor variables in Model 2. There is more to understanding collaboration beyond considering only the disciplines involved.

**Table 3:** Regression results predicting the extent of collaboration (using standardized regression coefficients)

	Model 1	Model 2
**Human capital**		
Research experience		0.896***
Research experience squared		−0.974***
Career advancement		0.079
**Cognitive aspects of science**		
Need for help of colleagues		0.100*
Agreement on quality research		0.142*
Research group composition		
Disciplinary–homogeneous		−0.069
Disciplinary–heterogeneous		0.003
Interdisciplinary		*base*
**Social aspects of science**		
Distrust in researchers		0.106*
Resource dependence		0.130*
**Control variables**		
Scientific discipline		
Mathematics	*Base*	*Base*
Sociology	0.059	−0.039
Physics	0.154**	0.038
Biotechnology	0.186***	0.022
Gender (female = 1)		0.016
Prior collaboration		0.124*
*F*	3.471**	3.780***
*R* ^2^	0.031	0.170

* $$p< 0.10$$, ** $$p < 0.01$$, *** $$p < 0.001$$

Our results show human capital variables had mixed impacts on collaboration. Both terms for research experience are statistically significant with the appropriate signs, supporting Hypothesis 1 (beginning researchers increase their involvement in collaborative research while the collaboration efforts decrease during the later stages of their careers). Hypothesis 2 about the collaboration being promoted as a means for advancing one’s career is not supported.

Cognitive aspects of research also have mixed impacts regarding the extent of collaboration. Having a need for collaboration, when there are research problems researchers cannot resolve promotes collaboration (Hypothesis 3) is supported. The significant effect for agreement on what constitutes quality research confirms Hypothesis 4. But the scope of collaboration in general does not depend on the disciplinary or inter-disciplinary composition of the project group, disconfirming Hypothesis 5.

Researchers spend more time collaborating when they do not trust researchers outside their own research unit. This significant result contradicts Hypothesis 6 about the negative impact of distrust on establishing and maintaining collaborative relations. This suggests a need to reexamine the link between trust and specific collaborative ties. We do this below. Collaboration is stimulated also by having to share resources to conduct research confirming Hypothesis 7. This is the second strongest predictor of collaboration.

### Explaining collaboration with different kinds of partners

The time researchers spend collaborating is the sum of their collaborative activities with different kinds of research partners. The foregoing analysis of the extent of collaboration assumed the underlying mechanism leading researchers to collaborate with others is tie-neutral, with the same model explaining the emergence of different kinds of collaborative ties. The results reported in Table 4 challenge this assumption by testing the hypotheses for different kinds of collaborative ties.

The inverse U-shaped relationship between research experience and extent of collaboration, as specified in Hypothesis 1, holds *only* for international collaboration. Clearly, researchers having longer research experience reduce their international involvement, most probably because long-distance collaboration is time consuming and difficult to combine with administrative and other engagements, and the productivity pay off is lower. It is also reasonable to think more experienced researchers form and lead their own research groups, leaving them less time for international collaboration.

Hypothesis 2, about the positive relationship between collaboration and career advancement, also holds *only* for international collaboration. Motivations regarding career advancement increase collaborative activity with international partners. In contrast to other studies, we cannot claim collaborating with colleagues from other Slovenian academic and non-academic organizations has a negative impact on researcher’s careers. While both coefficients are negative they are not significant.

Consistent with hypothesis 3, needing help from colleagues promotes collaboration, but again *only* with international partners. In small countries, such as Slovenia, researchers working in very specialized fields of work are more likely to collaborate with colleagues elsewhere.

Shared agreement about quality work (Hypothesis 4) has a significant impact on collaboration in general, as well as on two specific forms of collaboration within the organizations. This interpersonal element plays a role in opting for collaboration when more instrumental concerns such as resource dependence are absent (see below).

The interdisciplinary nature of research projects promotes collaboration with colleagues in other academic organizations supporting Hypothesis 5 to a limited extent. Collaborative research teams within the same organization and with non-academic organizations are as often interdisciplinary as disciplinary including various sub-specialties. Very differently, international collaboration is disciplinary. In the international context researchers tend to work together with colleagues from their narrow field of expertise.

**Table 4:** Regression results for collaboration with different partner types (using standardized regression coefficients)

	Same research center	Same organization	Other academic organization	Non-academic organization	International partners
**Human and social capital**					
Research experience	0.295	0.072	0.055	0.118	0.791***
Research experience squared	−0.278	−0.189	−0.006	−0.072	−0.756***
Career advancement	0.07	−0.07	−0.095	−0.08	0.128*
**Cognitive aspects of science**					
Need for help from colleagues	0.005	−0.091	−0.009	0.008	0.173**
Agreement on quality research	0.116*	0.103*	−0.012	−0.011	0.045
Research group composition					
Disciplinary– homogeneous	−0.009	−0.167*	−0.303***	−0.121*	0.116*
Disciplinary– heterogeneous	0.051	0.038	−0.116*	−0.077	−0.51
Interdisciplinary	*Base*	*Base*	*Base*	*Base*	*Base*
**Social aspects of science**					
Distrust in researchers	0.112*	0.04	0.049	0.044	0.023
Resource dependence	−0.002	−0.057	0.132*	0.183**	0.122*
**Control variables**					
Scientific discipline					
Mathematics	*Base*	*Base*	*Base*	*Base*	*Base*
Sociology	0.081	0.023	0.094	−0.051	−0.143*
Physics	0.170*	−0.098	−0.07	−0.035	−0.021
Biotechnology	0.211**	−0.059	−0.034	0.271*	−0.150*
Gender (female = 1)	−0.024	0.162*	−0.065	−0.078	−0.140*
Prior collaboration	0.101	0.056	0.055	0.103*	−0.023
*F*	1.671	2.749	2.676	3.866	4.611
*R* ^2^	0.083	0.13	0.127	0.173	0.2

* $$p< 0.10$$; ** $$p < 0.01$$, *** $$p < 0.001$$

Variables measuring collaboration with partners from other academic and non-academic organizations are log-transformed

The results in Table 4 support hypotheses regarding the social aspects of the organization of science. The distrust of other researchers promotes collaboration within research centers. Consistent with Hypothesis 6, a high level of collaborative engagement within the research group accompanies intense competition with and distrust of those outside the group. However, distrust of colleagues in other research centers and organizations does not have negative effect on establishing collaboration with them: this suggests high within-unit collaboration ought not be understood as a withdrawal but rather as an intense mobilization of internal human resources to improve the center’s competitive advantage. Also, considering researchers’ motivations, it might be that the negative effects of competition and distrust on the collaboration outside the research center are out-weighed by the positive effects of seeking competitive advantages by working with prominent colleagues and teams, resulting in non-significant effect for distrust.

Resource dependence does promote collaboration with colleagues in other academic and non-academic organizations supporting Hypothesis 7. Pooling resources to be able to conduct research is a less important factor when it comes to international collaboration or collaboration within same organization.

Regarding the control variables, gender affects collaborative work with colleagues working in different organizational domains and other countries. While there are no gender differences in the level of collaboration in general, women are more likely to connect with members of other research units and departments within same organization: the gender of researchers is important for maintaining intra-organizational networks where they bridge sub-disciplinary boundaries. At the same time, women are less likely to collaborate with international partners.

## Summary and conclusion

Considering both survey and bibliometric data adds complementary but contrasting information regarding collaboration. This comparison, at least for Slovenian researchers, reveals bibliometric data as underestimating collaboration levels in some scientific disciplines, most notably for mathematics and sociology, compared to biotechnology and physics. For biotechnology, where research collaboration is assumed to consume most of researchers’ time, researchers report they spend about two-thirds of their time collaborating with others. The consistency of our survey results with the bibliometric studies regarding collaboration patterns in different scientific disciplines can be viewed as a sign of the quality of the survey data. Despite the arguments claiming survey data being highly unreliable, we could account for a substantial portion of the variation of various measures of collaboration.

The extent of collaboration across different types of partners can be explained by three sets of factors: (1) individual human capital (2) cognitive aspects of research fields; and (3) social aspects of these fields. Regarding individual human capital: the temporal collaboration pattern over research careers has the expected inverted U-shape, but the motivation of career advancement through collaboration is not significant. This factor had differing impacts on collaboration with different types of research partners. Both the cognitive characteristics and social organization of research fields have strong impacts on overall collaboration.

Higher levels of collaboration with partners in the same research unit characterize both biotechnology and physics due, most likely, to laboratory work. Higher collaborative levels within research units is promoted also by coordinated mobilization of human resources within the research group which is intimately related to competition between researchers, and facilitated by the agreement on quality standards.

Intra-organizational collaboration tends to be sustained more by women with greater collaboration levels with colleagues across sub-disciplinary areas, an effort also facilitated with the agreement on the quality standards.

When researchers need to pool resources to conduct large scale and comprehensive research projects they turn to colleagues in other domestic, academic and non-academic, organizations with the latter being especially important. They also collaborate with colleagues from other academic organizations and their research centers to pursue interdisciplinary research. Interdisciplinarity is less of a stimulus for collaboration with non-academic organizations where researchers combine knowledge and practical expertise within the disciplinary and interdisciplinary research environment alike.

Although the collaboration with researchers in other organizations helps to realize the researchers’ ambitions for involvement in research projects to work on specific research problems, it contributes little towards faster promotions. Only collaboration with international colleagues has a positive impact on this aspect of researchers’ careers. Researchers dependent on visible international publications, tend to collaborate more with international colleagues. These contacts tend to be narrowly disciplinary. Yet collaboration with international partners tended to decline for researchers having more years of research experience.

Several policy implications stem from of our results. First, it is crucial for research policy in small countries to support the internationalization of research networks of its scientists. These networks are crucial at the time when standards for promotion and criteria for acquiring public research funds place higher emphases on academic excellence. To this end, researchers are more actively engaged with international collaboration when the length of their research careers is between 10 and 25 years. Women are significantly less involved in international collaboration than men. The latter calls for the policies targeted especially to women scientists facing the challenge of balancing the work and family. Second, having an equal representation of men and women in science is critical since women bring a potential for collaboration across intra-organizational divisions. Third, competition for resources related to distrust of others need not jeopardize large-scale collaboration when it promotes stronger local research units and centers. Finally, regardless of the kind of collaboration, creating common standards of quality stimulates collaboration. This is especially important in research fields where collaboration is less a result of instrumental considerations (as in sociology, for example) but depends on various facilitating factors including agreement on quality.

This summary of our results suggests strongly that studying collaboration with different kinds of collaboration partners merits further attention. While some of our results may have relevance only in small science systems, especially regarding the international collaboration and its effect on the career on researcher careers, contemporary processes of science promote different kinds of collaboration patterns for creating scientific knowledge. These include: scientific *competition* creating internally connected and collaborative research centers; common *standards of quality* promoting within-organization collaboration; *resource dependence* and * interdisciplinary research* stimulating collaborations between researchers in different organizations; knowledge *specialization* leading to extensive international collaborations, albeit in narrowly disciplinary foci. These patterns require further examination by studying and comparing more scientific disciplines, including the humanities. Having deeper understandings of multiple collaboration–specific mechanisms can help create research policies aimed at promoting even more productive collaboration.

## Appendix 1

In the last 12 months, what proportion of your research time was spent working alone and collaborating with specific partners? (percentages)

- Working alone.
- Collaborating with colleagues from the same research unit (research center or group).
- Collaborating with colleagues from the same organization, but not the same research unit.
- Collaborating with colleagues from other Slovenian academic institutions (universities and institutes).
- Collaborating with Slovenian colleagues from non-academic institutions (business sector, public sector, non-governmental sector).
- Collaborating with colleagues from abroad.

**Table 5:** Descriptive statistics for the extent of collaboration and collaborating with different kinds of partners

Variables	*N*	Mean	(SD)	Min–max	Skewness
Extent of collaboration	325	61.91	(25.22)	0–100	−0.375
Collaborating with:					
Research center	325	24.44	(19.86)	0–100	0.848
Same organization	325	10.61	(14.48)	0–100	2.634
Another academic organization	325	7.71	(12.92)	0–100	3.465
Non-academic organization	325	1.80	( 4.96)	0–100	3.977
International partners	325	15.03	(17.41)	0–100	1.741
Log another acad. organization	325	−0.15	( 2.42)	−2.30 to 4.61	0.570
Log Non-academic organization	325	−1.50	( 1.72)	−2.30 to 3.68	1.749

The variable extent of collaboration is approximately normally distributed. Variables measuring collaboration with different research partners are right-skewed. When the skewness statistics were larger than 3, we logtransformed the variables. These variables are collaborating with researchers from academic and non-academic organizations (Table 5).

## Appendix 2

See Table 6.

**Table 6:** Correlation matrix

	Extent of collaboration	Research unit	Research organization	Academic organization	Non-academic organization	International partners	Gender male = 0
Research unit	0.486***						
Research organization	0.270***	−0.203***					
Academic organization	0.245***	−0.055	0.057				
Non-academic organization	0.247***	0.135**	0.030	0.273***			
International partners	0.366***	−0.134**	−0.165**	−0.171**	−0.106*		
Gender male = 0	0.099	0.085	0.207***	0.075	0.005	−0.172**	
Research exper. squared	−0.107*	−0.038	−0.100*	−0.033	0.023	0.028	−0.204***
Collaboration experience	0.161**	0.132*	0.072	0.029	0.053	0.001	0.088
Knowledge codification	0.048	0.083	−0.085	0.115*	0.052	−0.061	−0.014
Agreemt. quality	0.133*	0.092	0.051	−0.018	0.004	0.129*	−0.019
Research problems	0.087	−0..015	−0.101*	−0.053	0.035	0.190**	−0.055
Trust in others	−0.065	−0.070	−0.037	0.021	−0.072	−0.054	−0.008
Career advancem.	0.181**	0.109*	−0.010	−0.029	−0.022	0.152**	0.104*
Resource depend.	0.266***	0.213***	−0.026	0.179**	0.285***	0.084	0.144**

	Research exper. squared	Collaboration experience	Knowledge codification	Agreement quality	Research problems	Trust others	Career advancem.
Research unit							
Research organization							
Academic organization							
Non-academic organization							
International partners							
Gender male = 0							
Research exper. squared							
Collaboration experience	0.119*						
Knowledge codification	−0.103*	0.060					
Agreemt. quality	0.113*	0.283***	0.176**				
Research problems	0.025	0.061	0.091	0.098			
Trust in others	0.006	0.234***	0.109*	0.234***	−0.041		
Career advancem.	−0.096	0.158**	0.074	0.223***	0.012	0.056	
Resource depend.	0.014	0.072	0.077	0.063	−0.028	−0.144**	0.284***

* $$p< 0.10$$, ** $$p <0.01$$, *** $$p < 0.001$$
